# Direct RNA sequencing mediated identification of mRNA localized in protrusions of human MDA-MB-231 metastatic breast cancer cells

**DOI:** 10.1186/1750-2187-8-9

**Published:** 2013-09-01

**Authors:** Kristine Raaby Jakobsen, Emilie Sørensen, Karin Kathrine Brøndum, Tina Fuglsang Daugaard, Rune Thomsen, Anders Lade Nielsen

**Affiliations:** 1 Department of Biomedicine, Aarhus University, Aarhus, Denmark; 2 Present address: Department of Molecular Biology and Genetics, Aarhus University, Aarhus DK-8000, Denmark; 3 Center for Integrative Sequencing, iSEQ, Aarhus University, Aarhus, Denmark; 4 Lundbeck Foundation Initiative for Integrative Psychiatric Research, iPSYCH, Aarhus University, Aarhus, Denmark

**Keywords:** Breast cancer, Metastasis, Boyden chamber, RNA localization, Protein localization

## Abstract

**Background:**

Protrusions of cancer cells conferrers a vital function for cell migration and metastasis. Protein and RNA localization mechanisms have been extensively examined and shown to play pivotal roles for the functional presence of specific protein components in cancer cell protrusions.

**Methods:**

To describe genome wide RNA localized in protrusions of the metastatic human breast cancer cell line MDA-MB-231 we used Boyden chamber based methodology followed by direct mRNA sequencing.

**Results:**

In the hereby identified group of protrusion localized mRNA some previously were described to be localized exemplified by mRNA for *Ras-Related protein 13* (*RAB13*) and *p0071* (*Plakophilin-4/PKP4*). For other transcripts, exemplified by mRNA for *SH3PXD2A/TKS5* and *PPFIA1/Liprin-α1*, only the corresponding proteins previously were described to have protrusion localization. Finally, a cohort of MDA-MB-231 protrusion localized transcripts represents novel candidates to mediate cancer cell subcellular region specific functions through mRNA direction to protrusions. We included a further characterization of p0071, an armadillo repeat protein of adherence junctions and desmosomes, in MDA-MB-231 and non-metastatic MCF7 cells including analysis of novel identified alternative spliced *p0071* mRNA isoforms. The results showed isoform and cell type specific *p0071* mRNA localization.

**Conclusions:**

Altogether, the presented data represents a genome wide and gene specific descriptive and functional analyses of RNA localization in protrusions of MDA-MB-231 metastatic cancer cells.

## Background

A metastasizing cancer cell is abridged to essential challenges: the ability to escape from the tumour and migrate to the blood vessels; survive in the circulation; make an entrance in another tissue; and the capability of surviving and growing in the new environment [1]. A metastasising cancer cell arising from epithelial tissue is characterized by the gain of mesenchymal properties involving increased motility in a transition that equals the epithelial-mesenchymal transition (EMT) seen during development [2]. Epithelial cells function as a barrier to establish and maintain an internal and an external environment and are immobile with a clear apico-basal polarity. Mesenchymal cells on the other hand are mobile, have more transient adhesive properties, are more responsive to extracellular attractants directing the movements, are capable of invading the basement membrane, and without apico-basal polarity [2]. Migration can occur as a response to variation of the stimulation from the extracellular environment, such as gradients of chemokines and extracellular matrix proteins. The cancer cell migration process is dynamic with attachments and detachments to the extracellular matrix resulting in movement [3]. This is mediated by cellular adhesion molecules such as integrins and cadherins and conformational cytoskeletal changes with resulting pseudopodial dynamics [4]. Integrins have different affinities for the extracellular matrix proteins and may activate and respond to different signalling pathways. In general, integrins have a high expression at the cellular leading edge and serve to stabilize pseudopodia through mediating contacts between the cytoskeleton and extracellular matrix (ECM) proteins [4,5]. The ability to form pseudopodial protrusions is closely related to cancer cell invasive and metastatic properties [6,7]. A core for pseudopodia formation is generated at the ventral surface of the cell by dendritic and bundled actin networks [8]. Actin polymerization and contractility are mediated by members of the RHO family of small GTP-binding proteins, including RAC, RHO and CDC42, activated by integrin interactions [9,10]. The actin core grows by elongation of actin bundles denoted filopodia [8]. If the actin dendritic network (lamellipodia) advances as fast as the actin bundles elongate, the structure of the elongated invadopodium appears as a microspike or roots of filopodia. Elongation of pseudopodia beyond 5 μm is permitted by the infiltration of microtubules. Intermediate filaments are entering in mature pseudopodia and have an impact only in later stages of invasion [8].

Asymmetric localization of RNA is involved in cell polarisation and pseudopodial formation [11,12]. RNA localization depends on motor protein movements on the microtubules and the actin filaments of the cytoskeleton [11,13]. Moreover, RNA localization depends on a dynamic interaction between *cis*-elements within the RNA and associated *trans*-factors forming localizing RNA protein complexes. A well-studied example is ZBP1 which is a RNA-binding *trans*-factor for RNA localization involved in generating apico-basal polarity and repressed in metastasizing breast cancer cells [14]. ZBP1 associates with the zip-code *cis*-element present in the 3′-UTR of the *β-actin* mRNA and mediates transport of the complex to the leading edge dependent on an intact actin cytoskeleton [14,15]. The RNA *cis*-elements for localization can either be directly sequence dependent as the zip-code or structural dependent. Several studies have addressed the identity of mRNA and protein with localization to pseudopodia in neurons, astrocytes, fibroblasts, and cancer cells [16-21]. Proteome and array based transcriptome analyses of metastatic tumour cell lines have resulted in the identification of pseudopodia localized proteins involved in pseudopodia formation, actin cytoskeleton dynamics, and cell migration and invasion [22].

The current knowledge concerning mRNA localization to metastatic cell pseudopodial protrusions is not yet fully comprehensive. We took advantage of a recently presented high throughput next generation sequencing method, direct RNA sequencing (DRS), which is a highly sensitive and low bias approach to identify and quantify total mRNA [23-25]. RNA localized in metastatic breast cancer cell pseudopodial protrusions was purified from the metastatic human breast cancer cell line MDA-MB-231 using a refined Boyden chamber assay [18,26-28]. Poly-adenylated mRNA molecules isolated from MDA-MB-231 cell protrusions and cell bodies were analyzed by DRS and protrusion localized mRNA determined. The presented procedure is an alternative method for identification of pseudopodial protrusion localized RNA and can contribute to broaden the overall understanding of the involvement of sub-cellular mRNA localization in cancer cell metastasis.

## Materials and methods

### Boyden chamber assay for cell protrusion isolation and DRS

Human breast cancer MDA-MB-231 and MCF7 cells were purchased from the American Tissue Culture Collection (ATCC). Before experimental procedures, cells were passaged once using 0.5% trypsin-EDTA (GIBCO). Cells were grown in DMEM (GIBCO) with 10% fetal calf serum (FCS), glutamine, penicillin, and streptomycin at 37°C in 5% CO_2_ humidified atmosphere [29].

A modified cell protrusion isolation method was used [26,28]. For the Boyden chamber assay we used 9.6 cm^2^ (6-well format) cell culture inserts (BD Falcon) with a 1 μm pore size uncoated polystyrene membrane. Membranes were coated with the ECM proteins collagen type-I (Sigma C7661) in a 10 μg/mL final concentration incubating at 37°C. for 2 h. Afterwards the membranes were directly transferred to a 6-well tissue culture dish containing serum free DMEM. Cells were serum starved overnight and detached by 0.5% Trypsin-EDTA, and the trypsin was inactivated by suspending cells in DMEM containing 10% FCS. Cells were pelleted and re-suspended in serum free medium, and 2 × 10^6^ cells were transferred to one membrane insert. Cell protrusions were allowed to grow through the membrane for 24 h before protein and RNA purification.

For RNA and protein purification inserts were washed in PBS and cell protrusions were harvested using a sharp cell scraper (Costar). The lower side of the membrane was scraped first and the cell scraper thoroughly washed in 1 ml TRI-Reagent (Sigma) for RNA or in 1 ml 1x loading buffer (Fermentas) for protein. It was carefully checked that the cell scraper was only used once on the membrane in order to avoid cell lysis by the TRI-Reagent or loading buffer, which could have resulted in contamination of the protrusion fraction of lysed cells from the upper-side. Cells on the upper side was subsequently scraped and transferred to TRI-Reagent or loading buffer. RNA was precipitated using 1 μg glycogen. Single RNA molecules were sequenced by DRS [24] using the Helicos Biosciences platform (Helicos Biosciences, Boston, MA). DRS was performed once with pools of RNA representing CF or PF from six independent Boyden chamber inserts. Equal amounts of RNA isolated from CF or PF were used for input in DRS. Bioinformatics raw data analysis and sequence alignment was made by Helicos Biosciences.

### RNA visualization and immunofluorescence

Total cytosolic RNA was visualized by ethidium bromide (EtBr) essential as previously described [28]. Briefly, MDA-MB-231 cells were prepared as described under Boyden Chamber assay. RNase control cells were made by treating membranes with 125 ng/μL RNase for 30 minutes. RNase was inactivated by vanadyl-ribonucleoside complex for 10 minutes. Membranes were washed twice with PBS, cells permeabilized in Triton X-100 0.5% for 10 min and subsequently washed with PBS. 1 μM EtBr in PBS was added for 15 min, cells washed twice with PBS and DAPI stained for 2 min. After washing in PBS, half of the cell material on the upper side of the membrane was wiped away. Membranes were sliced and in both orientations fixated in a drop of Prolong GOLD mounting media (Invitrogen) in a petri dish and analysed by microscopy.

For immunofluorescence analysis MDA-MB-231 or MCF7 cells were grown to ~70% confluence on 0.17 mm thick cover slips (Marienfeld), fixed in 4% paraformaldehyde in PBS (Electron Microscopy Sciences) for 20 min at room temperature, and permeabilized in 0.5% tritonX-100 (Sigma). Cells were incubated with primary antibody dissolved in blocking buffer for 1 h at room temperature, and were then treated with secondary antibody in blocking buffer for 45 min at room temperature. Cell nuclei were stained with DAPI and the coverslips were mounted. In immunofluorescence analysis of Boyden chamber membranes the cell bodies were removed from one half of the upper-side of the membrane. Membranes were incubated with antibodies as described above. Instead of cutting out the membrane and mounting, the Boyden chamber inserts were kept in PBS for visualization. For immunofluorescence rabbit anti-α-tubulin 1:500 (Rockland), anti-p0071 1:10 (Progen) and secondary antibody Alexa 488 conjugated goat anti-rabbit IgG 1:2000 (Invitrogen), Alexa 555 conjugated donkey anti-rabbit IgG 1:2000 (Invitrogen) or Alexa 555 conjugated goat anti-mouse IgG 1:2000 (Invitrogen) was used. All images for IF were made on a Zeiss axiovert 200 m microscope, with a plan apochromatic 63x 1.4 NA objective, a HBO 100 W mercury light source, and a CoolSNAP-HQ cooled CCD camera (Photometrics) operated by MetaMorph®. We took z-stacks with 20 sections 0.2 μm step size and 500 ms exposure. For the shown immunofluorescence images a single, best in focus, 2D image was selected from a 20 sections z-stack.

### Reverse transcriptase, PCR and RT-qPCR

cDNA was made from total RNA by iScript cDNA synthesis kit (Bio-Rad) containing both random hexamer and oligo(dT) primers using 1 μL purified total RNA solution per reaction. PCR was performed under standard conditions [28]. Quantitative real time PCR (RT-qPCR) was performed using SYBR Green 480 master mix (Roche), and reactions were run on a Roche Lightcycler™ 480, with a primer annealing temperature of 58°C. PCR products were verified by gel electrophoresis and melting curve peaks. Primer sequences are shown in Additional file 1: Table S1. RT-qPCR amplifications were made in triplicates for each gene and the Ct values were converted into linear values using the X_o_ method [30]. Student’s 2-Tailed t-test was performed accordingly to (http://studentsttest.com). The localization ratio was determined as the ratio between the mean expression in the cell body fraction and the protrusion fraction. RT-qPCR experiments to verify DRS results were performed on Boyden chamber purified RNA samples purified and processed independently of the RNA samples used for DRS. For *p0071* mRNA isoform detection the primer sequences are shown in Additional file 1: Table S1.

### Western blotting

Proteins were separated by SDS-PAGE in a 4-15% gradient polyacryl amide gel (Bio-Rad). Proteins were transferred to a nitrocellulose membrane which was cut in two parts. The following primary antibodies were used for protein detection: Anti Histone H3 (1:10000, Rabbit, Abcam ab1791, 19 kDa); Anti α-Tubulin (1:5000, Rabbit, Rockland 600-401-880, 51 kDa), Anti β-Actin (1:2500, Rabbit, Sigma A2103, 42 kDa); Anti Zeb1 (1:500, Rabbit, Sigma HPA027524, 200 kD); Anti ANP32B (1:100, Rabbit, Sigma SAB4500125, 30 kDa). Antibody procedures and detection by horse radish peroxidase (HRP) conjugated antibody (Dako, 1:10000) were performed as previously described [28,29].

## Results and discussion

### RNA isolation from MDA-MB-231 cell protrusions

**Figure 1:**
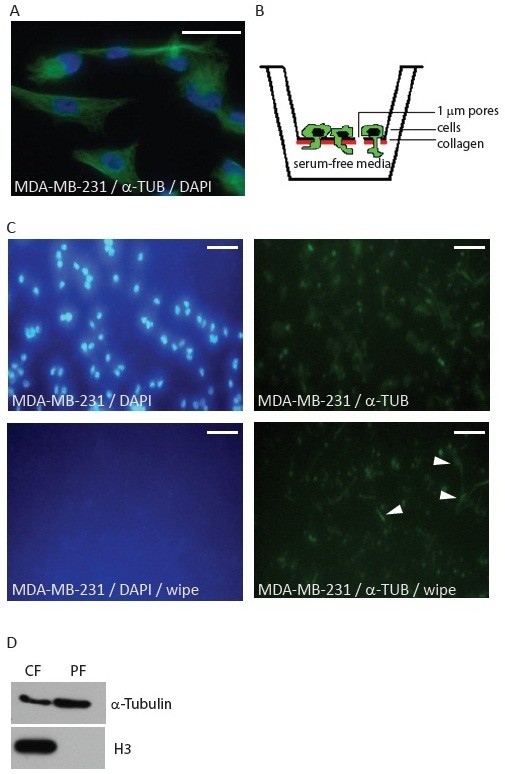
**Trans-migration of MDA-MB-231 protrusions in a modified Boyden chamber assay. (A)** Immunofluorescence image showing the typical morphology of human breast cancer MDA-MB-231 cells. The cellular cytoplasm was stained for α-Tubulin (α-Tub, green) and nuclei by DAPI (Blue). Scale bar 20 μm. **(B)** Schematic drawing showing the principle of the Boyden chamber set-up for isolation of migrating protrusions. Cells were grown on a 1 μm pore size micro porous membrane allowing trans-migration of only the cell protrusions. The lower side of the membrane is coated with ECM protein. **(C)** Protrusions of MDA-MB-231 cells migrated through a 1 μm micro porous membrane after 24 h assay time. α-Tubulin (green) and DAPI staining (blue) is shown without and after wipe (wipe) to remove cellular material present on the upper side of membranes. Examples of protrusions present on the Boyden chamber membrane lower side are indicated by arrowheads. Scale bar 100 μm. **(D)** Histone H3 is absent from the Boyden chamber lower side protrusion fraction. Western blotting was performed with histone H3 antibody and α-Tubulin antibody on cell body fraction (CF) and protrusion fraction (PF) protein samples originating from pooled material from three independent Boyden chambers.

To characterize mRNA localized in metastatic breast cancer cell protrusions we used the human breast cancer cell line MDA-MB-231 with bone metastatic potential and a mesenchymal like elongated morphology (Figure 1A). To isolate RNA from protrusions we took advantage of a modified Boyden chamber cell fractionation method developed to separate cell protrusions from cell bodies [28]. The Boyden chamber consists of two compartments separated by a micro porous membrane through which cells can migrate (Figure 1B). By applying membranes with a sufficiently small pore size, it is possible to restrict membrane trans-migration to only the thin cell protrusions and not the entire cell [28]. Cell protrusion *trans*-migration is stimulated by ECM protein coating of the membrane lower side. MDA-MB-231 cells were cultured until 80% confluence in serum free medium before transfer to the Boyden chamber. Verification of protrusion migration, and not cellular *trans*-migration, was made by a total cytoplasm fluorescent staining of the omnipresent cytoplasmic protein α-Tubulin and staining of nuclei with DAPI. Before imaging, cells on one half of the upper side of the membrane were carefully removed using a cotton stick (Figure 1C). Membrane upper side wiping removed all DAPI staining in accordance with the presence of no *trans*-migrated cell nuclei to the membrane lower side. α-Tubulin staining was still visible on the lower membrane side indicating the presence of the protein in protrusions (Figure 1C). Western blot analyses were performed for protein extracts isolated from the cell bodies present on the upper membrane side (cell body fraction, CF) and from the protrusions (protrusion fraction, PF) from the membrane lower side. Histone H3 was only detectable in the CF supporting lack of cellular *trans*-migration (Figure 1D). Western blot analysis of α-Tubulin was used to control for equal protein amounts in the CF and PF extracts (Figure 1D).

**Figure 2:**
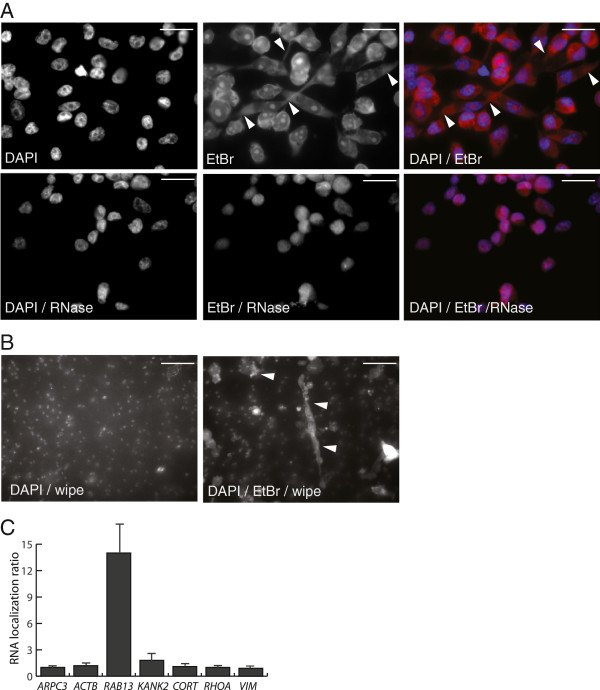
**RNA in MDA-MB-231 protrusions. (A**-**B)** Ethidium bromide (EtBr) mediated visualization of RNA in protrusions of MDA-MB-231 cells grown in Boyden chamber. **(A)** RNase treated cells (+ RNase) are shown for control of RNA specific EtBr staining. The left panels show DAPI staining, central pictures EtBr staining and merged pictures are shown in the right panels. Arrowheads exemplify EtBr staining in cell protrusions. Scale bar 20 μm. **(B)** EtBr mediated visualization of RNA localization in protrusions migrated through the 1 μm pore size membrane in the Boyden chamber. The cell bodies on the upper side of the membrane seen in **(A)** were removed by wiping and DAPI staining was performed in parallel to the pictures shown in **(A)** (left panel). EtBr staining was used to visualize cell material at the membrane lower side (arrowheads, right panel). Exposure times were increased compared to **(A)** visualizing also the 1 μm membrane pores. Scale bar 20 μm. **(C)** Determination of the relative RNA localization ratio in MDA-MB-231 protrusions by RT-qPCR analyses of candidate genes. The RNA localization ratio was calculated as the relative expression level in the protrusions fraction compared to the cell body fraction. The localization ratio of RNA in protrusions was normalized to the RNA localization ratio of *ARPC3* given the value 1.

Next we analyzed Boyden chamber grown MDA-MB-231 cell protrusions for the presence of RNA. Total RNA and DNA amounts were stained by ethidium bromide (EtBr) in MDA-MB-231 cells present at the membrane upper sides (Figure 2A). Cell protrusions displayed a diffuse signal which disappeared after treatment with RNase free of DNAse activity (Figure 2A). This supported the presence of RNA in MDA-MB-231 cell protrusions. We note that mitochondrial DNA was envisaged also to be present in the cell protrusions but this was not monitored at the given assay conditions. We next examined for RNA content in cell protrusions migrated through the Boyden chamber membrane. The cell bodies were wiped away from the upper membrane side and the cell material present on the membrane lower side stained with EtBr. This showed the presence of EtBr staining, and thus RNA, in both minute and elongated MDA-MB-231 cell protrusions present on the lower side of the membrane (Figure 2B).

For RNA isolation MDA-MB-231 cell protrusions were separated from cell bodies by the Boyden chamber assay and total RNA from PF and CF was isolated for downstream analysis. Material was sampled from 6 independent Boyden chambers each containing 2 × 10^6^ cells. By quantitative real-time PCR (RT-qPCR) we monitored RNA quantity and quality by examining RNA previously determined to localize in MDA-MB-231 cell protrusions. RT-qPCR was performed on cDNA made from equal RNA concentrations from CF and PF. We included primers against *β-Actin* (*ACTB*), *ARPC3, RAB13*, *KANK2/ANKRD25*, *RHOA*, *Vimentin* (*VIM*), *GAPDH* and *Cortactin* (*COR*). *RAB13* and *KANK2* mRNA were in a previous study shown to be localized in protrusions of NIH3T3 cells [16]. RHOA, COR and ACTB are all involved in the dynamics of the actin skeleton, VIM is an intermediary filament protein with an expression correlating with the mesenchymal phenotype and is up-regulated during EMT, and *ARPC3* and *GAPDH* represents housekeeping genes. *ARPC3* mRNA was previously determined to have uniform cytoplasmic localization in mouse astrocytes and NIH3T3 cells and chosen as reference gene [16,28]. We observed localization for *RAB13* (14-fold) and *KANK2* (2-fold) mRNA compared to the used normalization control *ARPC3* mRNA whereas no localization was observed for *VIM*, *COR*, *RHOA* and *ACTB* (Figure 2C). Altogether the RT-qPCR analyses pointed towards that RNA isolated from MDA-MB-231 protrusions is in a quality and quantity which allows for further biochemical characterization.

### Direct RNA sequencing (DRS) of MDA-MB-231 protrusion and cell body mRNA

Previous transcriptome analysis of Boyden chamber isolated RNA from cancer cells have used microarray based methods [22]. Recent reports have shown reproducible NGS analysis by DRS using minute RNA quantities [23-25]. In the DRS procedure cDNA synthesis and amplification are evaded and NGS analysis can be performed directly on poly-adenylated RNA. MDA-MB-231 cells grown in Boyden chambers as described above and RNA isolated from CF and PF RNA analyzed by DRS using equal amounts of the RNA from the two fractions. The resulting output of 36 bases average size sequences were mapped to a 2 kb region surrounding the distal 3′ end of 36296 annotated poly-A transcripts. The total number of mapped sequences for CF RNA was 994212 and for PF RNA 1270262. The similarity in the number of mapped sequences is a reflection of the comparable amount of starting RNA material used for DRS. The number of sequences for each mRNA identified by DRS was normalized to represent the number mRNA transcripts per million total sequence reads (tpm). The normalization value for CF mRNA was 1.0058 and for PF mRNA 0.7872. The thereby calculated tpm values represent the relative abundance of a given mRNA in the total population of DRS identified mRNA molecules. For the subsequent analyses we only included mRNA with ≥ 5 tpm in both the CF and PF to avoid insignificantly results in ratio calculations due to putative stochastic fluctuations in numbers determined from the very low tpm range. The number of mRNA having ≥ 5 tpm in both CF and PF was 8195 (Additional file 2: Table S2). We next determined the relative mRNA localization ratio in PF compared to CF. RNA localization ratios for mRNA were calculated as the ratio between the tpm value in PF and CF. The absolute value of the mRNA localization ratio depends on cell morphology and experimental settings. However, the hierarchical order of localization ratios is emphasized to be relative independent of these variables. *ARPC3* mRNA was determined to have a localization ratio of 1.379 and we have in the following used the assumption that *ARPC3* represents a non-localized mRNA [16]. We subsequently used the conservative cut-off value that the localization ratio should be at least 1.6 to define a mRNA to have localization in protrusions. By using this cut-off value of 1.6 in total 709 mRNA were identified to be localized in MDA-MB-231 cell protrusions (All localized mRNA are shown in Additional file 3: Table S3). The 100 most protrusion localized mRNA are shown in Table 1. We note that a localization ratio > 1.6 for a given mRNA type is not equivalent to the presence of this mRNA specifically within protrusions but reflects the relative localization in protrusions compared to cell bodies. Thereby, protrusion localization of a partial fraction of the total mRNA amount for a given mRNA type is sufficient to obtain a localization ratio > 1.6.

**Table 1:** **MDA**-**MB**-**231 protrusion localized mRNA**

**Accession number**	**Name**	**tpm CF**	**tpm PF**	**Loc. ratio**
NM_002870	RAB13	12.07	303.09	25.11
NM_006617	NESccpr	7.04	168.47	23.93
NM_001142964	C22orf46	9.05	133.83	14.78
NM_015049	TRAK2	57.33	527.45	9.20
NM_021202	TP53INP2	14.08	120.45	8.55
NM_001128128	ZEB1	39.23	329.85	8.41
NM_014631	SH3PXD2A	56.33	455.02	8.08
NM_152793	C7orf41	5.03	40.15	7.98
NM_001165966	PITPNM3	12.07	90.53	7.50
NM_014945	ABLIM3	28.16	210.19	7.46
NM_006141	DYNC1LI2	34.20	243.26	7.11
NM_002360	MAFK	21.12	148.00	7.01
NM_001161576	SAMD4A	69.40	467.62	6.74
NM_001810	CENPB	29.17	190.51	6.53
NM_001007189	C5orf53	30.17	184.21	6.11
NM_005275	GNL1	11.06	62.19	5.62
NM_006401	ANP32B	94.55	526.66	5.57
NM_145166	ZBTB47	12.07	66.13	5.48
NM_005870	SAP18	43.25	229.09	5.30
NR_024278	LOC646762	7.04	37.00	5.26
NM_001099650	GXYLT1	5.03	25.98	5.17
NM_013321	SNX8	56.33	285.77	5.07
NM_020904	PLEKHA4	5.03	25.19	5.01
NM_003628	PKP4	18.10	88.96	4.91
NM_001160184	PLEKHN1	9.05	42.51	4.70
NM_001166110	PALLD	61.36	283.41	4.62
NM_005863	NET1	338.96	1548.50	4.57
NM_001164407	TLCD2	6.03	26.77	4.44
NM_014389	PELP1	9.05	40.15	4.44
NM_201379	PLEC	30.17	133.83	4.44
NM_004050	BCL2L2	79.46	351.90	4.43
NM_032409	PINK1	179.04	784.09	4.38
NM_001312	CRIP2	42.24	185.00	4.38
NM_178313	SPTBN1	6.03	25.98	4.31
NM_006371	CRTAP	8.05	33.85	4.21
NM_003626	PPFIA1	86.50	351.11	4.06
NM_001185078	ARHGDIA	40.23	162.96	4.05
NM_203378	MB	6.03	24.40	4.04
NM_007343	PRSS3	40.23	162.17	4.03
NM_014325	CORO1C	84.49	330.64	3.91
NM_033549	TRIM41	22.13	83.45	3.77
NM_001001551	C9orf103	5.03	18.89	3.76
NM_207343	RNF214	7.04	25.19	3.58
NM_001130036	PLEKHB1	9.05	32.28	3.57
NM_001136137	RPL28	1327.68	4629.75	3.49
NM_001007073	RPL32	5.03	17.32	3.44
NM_024299	PPDPF	69.40	237.75	3.43
NM_017712	PGPEP1	51.30	174.77	3.41
NM_001130012	SLC9A3R2	236.37	802.20	3.39
NM_174971	ST3GAL3	7.04	23.62	3.35
NM_005831	CALCOCO2	11.06	37.00	3.34
NM_181718	ASPHD1	12.07	40.15	3.33
NM_002084	GPX3	77.45	256.64	3.31
NM_138392	SHKBP1	27.16	89.75	3.31
NM_024535	CORO7	5.03	16.53	3.29
NM_004834	MAP4K4	20.12	65.34	3.25
NM_016533	NINJ2	8.05	25.98	3.23
NM_002889	RARRES2	16.09	51.96	3.23
NM_024112	C9orf16	80.47	259.79	3.23
NM_145637	APOL2	16.09	51.17	3.18
NM_001128303	C13orf31	21.12	66.92	3.17
NR_001593	RPL18AP3	6.03	18.89	3.13
NM_019592	RNF20	10.06	31.49	3.13
NM_080677	DYNLL2	43.25	133.83	3.09
NM_058163	TSR2	38.22	117.30	3.07
NM_001127396	STXBP2	52.30	159.02	3.04
NM_015044	GGA2	8.05	24.40	3.03
NM_001171888	FGFR1OP2	7.04	21.26	3.02
NM_054012	ASS1	7.04	21.26	3.02
NM_002375	MAP4	180.04	542.41	3.01
NM_181509	MAP1LC3A	12.07	36.21	3.00
NM_033257	DGCR6L	12.07	36.21	3.00
NM_015683	ARRDC2	48.28	143.28	2.97
NM_001687	ATP5D	70.41	208.62	2.96
NM_015383	NBPF14	30.17	87.38	2.90
NM_001195259	LOC645781	9.05	25.98	2.87
NM_002085	GPX4	331.92	947.84	2.86
NM_003755	EIF3G	214.24	606.96	2.8
NM_181526	MYL9	44.26	125.17	2.83
NM_006166	NFYB	5.03	14.17	2.82
NM_021630	PDLIM2	5.03	14.17	2.82
NM_031286	SH3BGRL3	657.81	1853.16	2.82
NM_001018	RPS15	63.37	177.92	2.81
NM_001170931	FOXO4	9.05	25.19	2.78
NM_001167880	LHPP	18.10	50.38	2.78
NM_014994	MAPKBP1	11.06	30.70	2.78
NM_001079812	DIAPH1	61.36	170.04	2.77
NM_032488	CNFN	6.03	16.53	2.74
NM_032421	CLIP2	6.03	16.53	2.74
NM_004395	DBN1	70.41	192.09	2.73
NM_002952	RPS2	232.34	631.37	2.72
NM_001136203	CCDC124	34.20	92.11	2.69
NM_012199	EIF2C1	5.03	13.38	2.66
NM_003249	THOP1	8.05	21.26	2.64
NM_054014	FKBP1A	16.09	42.51	2.64
NM_005528	DNAJC4	19.11	50.38	2.64
NM_057159	LPAR1	58.34	153.51	2.63
NM_015493	KANK2	117.68	308.60	2.62
NM_144497	AKAP12	6.03	15.74	2.61
NM_001184942	C9orf25	12.07	31.49	2.61

The 100 most localized mRNA are shown with their hierarchical order of the mRNA localization ratio (Loc ratio). Expression values are shown as transcripts per million reads (tpm) from cell body fraction (CF) and cell protrusion fraction (PF).

**Figure 3:**
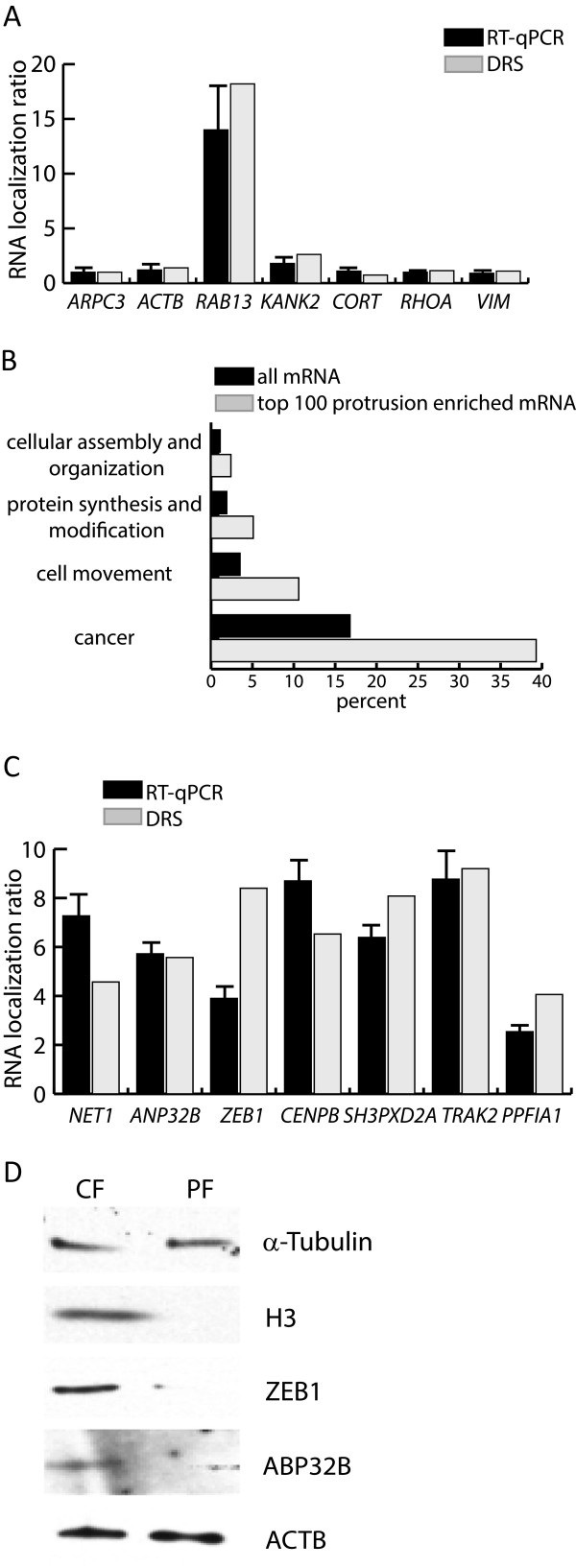
**DRS transcriptome analysis of purified RNA from MDA-MB-231 protrusions. (A)** Comparison of DRS and RT-qPCR analyses. RNA localization ratios were normalized to *ARPC3* mRNA given the value 1. **(B)** Annotation analysis of the 100 most MDA-MB-231 protrusion localized mRNA compared to the total group of mRNAs expressed with ≥ 5 tpm. Annotations were made using the IPA Ingenuity platform. **(C)** RT-qPCR confirmation analysis of selected MDA-MB-231 protrusion localized RNA identified by DRS. RNA localization ratios are normalized to *ARPC3* mRNA. **(D)** Western blot analysis. Western blotting was performed on pooled material from three independent Boyden chamber experiments representing cell body fraction (CF) and protrusion fraction (PF). α-Tubulin Western blot analysis was used to equalize the loaded protein amounts. Subsequent western blot analyses were performed with antibodies for Histone H3, ZEB1, ANP32B and ACTB.

We next compared the mRNA localization results from the DRS with RT-qPCR data (Figure 3A). Following normalization for *ARPC3* mRNA protrusion localization which was normalized to value 1 in both RT-qPCR and DRS we note very concordant results concerning mRNA localization by the two methods (Figure 3A). By DRS *RAB13* mRNA was identified as the most localized mRNA (ratio 25.1). This result was in concordance with the RT-qPCR result (Figures 2B and 3A) and with previous studies showing a significant *RAB13* mRNA localization in both astrocyte and fibroblast protrusions [16,28]. RAB13 is a member of the RAB family of GTPases and involved in neurite outgrowth through the process of filopodia formation [31]. We also note verification of DRS mRNA localization by RT-qPCR analysis for several other mRNA (Figure 3A and 3C). Thus, DRS and the subsequent RT-qPCR verification points that the Boyden chamber assay combined with DRS will be an applicable approach to search for protrusion localized RNA on a genome wide scale.

### Annotation analysis of MDA-MB-231 protrusion localized mRNA

We selected the top 100 most protrusion localized mRNA for annotation analysis using the IPA Ingenuity online platform. For reference we also analyzed a list of all transcripts determined from DRS with ≥ 5 tpm. The most striking outcome was 4 functional pathways overrepresented in the group of protrusion localized mRNA: Cellular assembly and organization; Protein synthesis and modification; Cell movement; and Cancer (Figure 3B). More surprisingly, functional pathway analyses and manual inspection pointed that the localized mRNA included several transcripts encoding nuclear proteins exemplified by *NET1* (ratio 4.6, NM_005863), *ANP32B* (ratio 5.6, NM_ 006401), *ZEB1* (ratio 8.4, NM_001128128) and *CENPB* (ratio 6.5, NM_001810) (Table 1 and Figure 3C). Transcripts for nuclear proteins were also identified in other studies describing protrusion localized mRNA and for example *NET1* mRNA was previously described to localize to the basement membrane in EMT during gastrulation [16,22,32]. In MDA-MB-231 cells NET1 controls focal adhesion kinase activation during cell movement and motility during ECM invasion in a RAC1 dependent extranuclear localization dependent manner [33]. ZEB1 protein is highly expressed in MDA-MB-231 cells and is a transcription factor involved in the regulation of breast cancer EMT, cell motility and metastasis [34]. An involvement of ANP32B and CENPB proteins in breast cancer cell migration and metastasis is to our knowledge not yet described but we note that elevated ANP32B expression is associated with poor prognosis in breast cancer [35]. RT-qPCR verified the mRNA localization in protrusions for *NET1*, *ANP32B*, *ZEB1* and *CENPB* (Figure 3C). Western blot analysis using CF and PF protein extracts normalized for α–Tubulin showed that in the PF ZEB1 and ANP32B were not detectable in agreement with in preference nuclear localization of the proteins (Figure 3D). We note that the minute amounts of protein material isolable from PF and the according high dilution of the CF sample to equalize for α–Tubulin expression level in the two samples hindered conclusive Western blot analysis of a large set of proteins assayed including for example NET1 and p0071 due to either an insufficient expression level or antibody sensitivity.

Shankar et al. previously presented the identification of protein and mRNA localizing to protrusions of different metastatic cancer cell lines including MDA-MB-231 cells [22]. mRNA localization was addressed by microarray studies of RNA purified from protrusions resulting in identification of 384 transcripts determined to have a protrusion localization ratio above the threshold value 1.6 [22]. Of these transcripts *Desmoyokin* (AHNAK nucleoprotein), *Septin-9* and *Calgizzarin* (S100A11) were shown to encode proteins involved in cell migration and invasion [22]. We note that by DRS we find mRNA localization in protrusions of *Desmoyokin* (mRNA localization ratio 1.62, NM_024060) and *Calgizzarin* (mRNA localization ratio 2.1, NM_005620) whereas *Septin-9* (ratio 1.1, NM_0011134914) mRNA localization was absent. The approximately 2-fold increase in the total number of identified protrusion localized mRNA by the hereby presented DRS approach could be a consequence of higher sensitivity and shows the importance of using different experimental approaches. Several of the mRNA species within the DRS mRNA list encode proteins with a described function in protrusions and cell migration processes. However, the participation of mRNA localization to confer the final protein localization has not been described for the majority of these transcripts. In the following we will give few examples of metastatic breast cancer relevant transcripts, which to our knowledge has not been attributed before with mRNA localization. The mRNA localization of this subgroup in cell protrusions was confirmed by RT-qPCR (Figure 3C). SH3PXD2A/TKS5/FISH (mRNA localization ratio 8.1, NM_014631) is a SH3 domain-rich protein which is an adaptor protein in clustering structural and enzymatic components of invadopodia [36,37]. SH3PXD2A function is required for invadopodia formation, for degradation of the extracellular matrix, and for cancer cell invasion [36,37]. The trafficking kinesin protein TRAK2 (mRNA localization ratio 9.2, NM_015049) is a mediator of axonal mitochondrial transport and is a kinesin adaptor for mitochondria at the tips of cellular processes [38]. PPFIA1/Liprin-α1 (mRNA localization ratio 4.1, NM_003626) adaptor protein is a regulator of cell edge dynamics during motility [39]. In MDA-MB-231 cells PPFIA1 is required for migration and invasion by mediating ECM degradation and the formation of lamellipodia and invadopodia [39].

### Characterization of *p0071* mRNA in MDA-MB-231 and MCF7 cells

**Figure 4:**
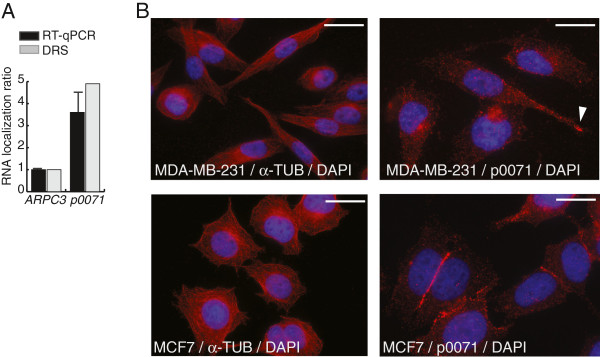
**Characterization of *p0071 *mRNA and protein localization. (A)** DRS and RT-PCR analysis of *p0071* mRNA localization in MDA-MB-231 cell protrusions. The expression level of *ARPC3* and *p0071* mRNA in PF and CF were determined by RT-qPCR and the *p0071* mRNA localization ratio normalized to *ARPC3* mRNA given the value 1. *p0071* mRNA was detected by primers recognizing all *p0071* mRNA isoforms (*pan-p0071*). **(B)** Protein localization of p0071 in MDA-MB-231 and MCF7 cells. α–Tubulin (left panels) and p0071 (right panels) were visualized by immunofluorescence analysis in MDA-MB-231 cells (upper panels) and MCF7 cells (lower panels). Cells were counterstained with DAPI to visualize nuclei. A representative p0071 staining in a cell protrusion is indicated by arrowhead. Scale bar 20 μm (left panels) and 10 μm (right panels).

p0071 (Pkp4/plakophilin-4) is a member of the armadillo family of proteins with δ-catenin/NPRAP/CTNND2 being the closest homolog [40-42]. p0071 and δ-catenin are located at desmosomes and adherence junctions playing a regulatory role in the dynamics of these intermediate filament and actin cytoskeletal plasma membrane assemblies [43-45]. Moreover, p0071 localize to the cytoplasm and acts as a positive regulator of the small GTPase RHOA whereas delta-catenin is a negative RHOA regulator [44,46]. p0071 protein interacts with numerous proteins including ERBB2IP/ERBIN, Presenilin-1, desmocollin-3A, desmoplakin, plakoglobin, and cadherins [42,43,47-50]. Recently, p0071 protein was shown to interact with Folliculin (FLCN), the product of the Birt-Hogg-Dube tumour suppressor gene and thereby regulating cell-cell adhesion and E-cadherin localization [51,52]. The *p0071* mRNA was shown to be localized in protrusions of NIH3T3 cells [16] and mouse astrocytes [28]. By DRS we identified MDA-MB-231 protrusion localization of *p0071* mRNA (ratio 4.9) (Table 1) which was verified by RT-qPCR (Figure 4A). By western blot analysis we were unable to evaluate the presence of p0071 protein in protrusions due to the low protein level isolated in from the PF in combination with low p0071 antibody sensitivity (data not shown).

p0071 is associated with different steps in carcinogenesis [40,43,53,54]. However, *p0071* mRNA localization has not been characterized in cancer cells and this was pursued in this study. Microtubule transport of *p0071* and *RAB13* mRNA to the cellular leading edge is dependent on Adenomatous polyposis coli (APC) protein which play a role in the anchoring of mRNA at the end of the microtubules [13]. The loss of APC, which like Folliculin is a tumour-suppressor protein, is an initiating event in colorectal cancer [13]. *APC* mRNA had a localization ratio of 1.4 in the DRS analyses which is similar to *ARPC3* mRNA indicating lack of protrusion localization. We also note the lack of *Delta-catenin* and *Folliculin* mRNA localization in MDA-MB-231 cell protrusions examined by DRS (ratio 0.5 and 0.7, respectively).

Localization analysis by immunofluorescence showed the presence of p0071 protein throughout the cytoplasm of MDA-MB-231 cells with mostly perinuclear localization but also localization in some cell protrusions (Figure 4B). We note that in the immunofluorescence analysis approximately 50% of the MDA-MB-231 cells displayed distinct protrusions and in approximately 50% of these p0071 protein was detectable (Figure 4B). α-Tubulin showed a uniform localization in MDA-MB-231 cells (Figure 4B). To determine the localization of p0071 protein in non-metastatic breast cancer cells we examined the MCF7 cell line with epithelial-like cell morphology (Figure 4B). In MCF7 cells p0071 protein localization was most prominent to cell-cell contact surfaces in agreement with previous descriptions of p0071 protein localization to desmosomes and adherence junctions of contacting epithelial cells (Figure 4B) [43]. The specificity of the p0071 antibody was verified by transfecting MDA-MB-231 cells with siRNA which resulted in p0071 depletion but no observable differences in protrusion forming capability and cell migration (data not shown).

**Figure 5:**
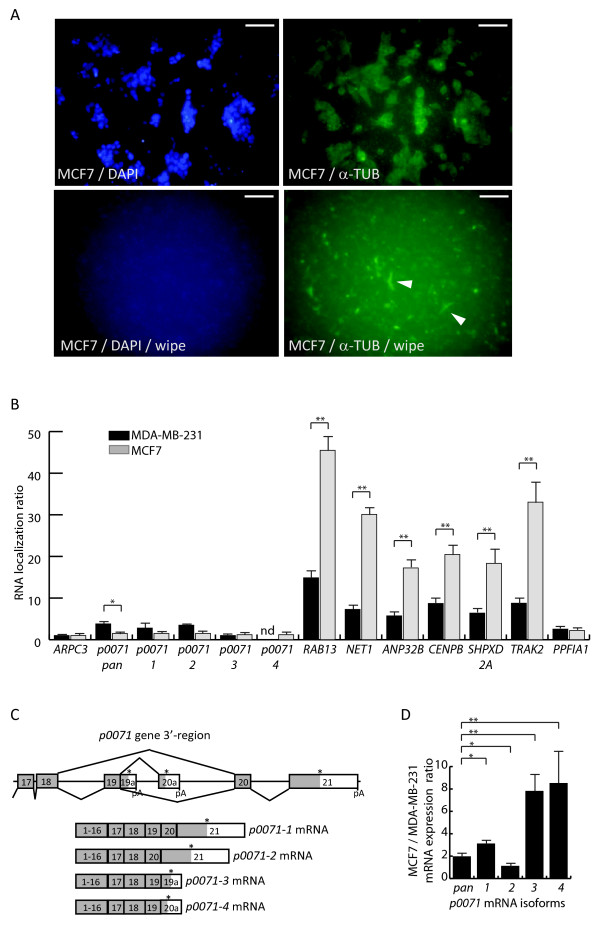
***P0071 *mRNA isoform localization in MDA**-**MB**-**231 and MCF7 cells. (A)** Boyden chamber analysis of MCF7 cells. Protrusions of MCF7 cells migrated through a 1 μm micro porous membrane after 24 h assay time. α-Tubulin (green) and DAPI staining (blue) is shown without and after wipe (wipe) to remove cellular material present on the upper side of membranes. Examples of protrusions present on the Boyden chamber membrane lower side are indicated by arrowheads. Scale bar 100 μm. **(B)** RT-PCR analysis of mRNA localization in MCF7 and MDA-MB-231 cells. The RNA localization ratio was calculated as the relative expression in the protrusion fraction compared to the cell body fraction. The localization ratio was normalized to the RNA localization level of *ARPC3* mRNA given the value 1. *Pan-p0071* indicates detection of *p0071* cDNA from exon 6 to exon 7. Student’s 2-Tailed t-test: *, p < 0.05; **, p < 0.005. **(C)** Alternative spliced *p0071* mRNA isoforms. Schematic drawing of *p0071* mRNA isoforms involving the 3′-region of the *p0071* gene detected at UCSC Genome Browser (NCBI36/hg18 assembly). pA indicates poly-adenylation signals. Exon 19a and exon 20a represents novel *p0071* exon sequences where exon 19a is a 3′-extension of exon 19. Alternative splicing is indicated above the exons and the consensus *p0071* splicing below. The four *p0071* mRNA isoforms are schematically shown with exons 1 to 16 only indicated. Asterisk above exons show translational stop codons. **(D)** Expression level analysis of *p0071* mRNA isoforms in MCF7 cells relative to MDA-MB-231 cells. Expression of *p0071* mRNA isoforms was detected by RT-qPCR, normalized to *GAPDH* expression, and the expression ratio between MCF7 and MDA-MB-231 cells calculated by division. RT-qPCR experiments were performed minimum three independent times and analyzed with Student’s 2-Tailed t-test: *, p < 0.05; **, p < 0.005.

Given the observed p0071 protein localization in MDA-MB-231 and MCF7 cells we next examined the *p0071* mRNA localization in MCF7 cells using the Boyden chamber approach (Figure 5A). We were able to isolate a lower amount of total RNA from MCF7 protrusions compared with MDA-MB-231 cells which we attributed to different morphologies of the two cell lines. RT-qPCR analysis revealed a significantly increased *RAB13* mRNA protrusion localization ratio in MCF7 cells compared to MDA-MB-231 cells (Figure 5B). This was also observed for *NET1*, *ANP32*B, *CENP*, *SHPXD2A* and *TRAK2* mRNA (Figure 5B). This could reflect a higher intrinsic mRNA localization capacity for these mRNA in MCF7 cells or be a consequence of the different morphologies of MDA-MB-231 and MCF7 protrusions. It can be envisaged that if an mRNA is localized to distal end of a cell protrusion a decrease in total protrusion material isolated due to a small protrusion size will result in a determined higher mRNA localization ratio. In MCF7 cells the protrusion fraction represents a smaller amount of the total cellular material than in MDA-MB-231 cells and as consequence mRNA localization ratios in general could be higher. Different from the increased MCF7 localization ratios for *RAB13*, *NET1*, *ANP32*B, *CENP*, *SHPXD2A* and *TRAK2* mRNA, the *p0071* mRNA localization ratio in MCF7 protrusions was decreased compared to MDA-MB-231 protrusions (Figure 5B). The decrease in *p0071* mRNA localization ratio in MCF7 protrusions was in accordance with an observed MCF7 p0071 protein localization to mostly cell-cell contact surfaces (Figure 4B).

Subcellular mRNA localization is mediated by a combination of mRNA *cis*-elements, as for example the Zip-code and G-rich sequences often residing in the 3′-UTR, which associates with *trans* acting localization factors [11,13]. We note that the mRNA/mRNP transport protein FMRP previously was described to target *p0071* mRNA [16,55,56]. Inspection of the *p0071* gene at UCSC Genome Browser (NCBI36/hg18 assembly) showed the presence of putative alternative splicing and poly-adenylation events consequently generating *p0071* mRNA isoforms with different coding regions and 3′-UTR sequences. Schematic description of the hereby examined four putative *p0071* mRNA isoforms is shown in Figure 5C. *p0071-1* (transcript variant 1) includes exon 19 and encodes a protein of 1192 amino acids. *p0071-2* (transcript variant 2) lacks exon 19 (length 129 base pairs) and as consequence results in an internal p0071 protein deletion of 43 amino acids with a resulting p0071 protein isoform of 1149 amino acids. The alternative poly-adenylated *p0071-3* and *p0071-4* mRNA isoforms include an extended exon 19 sequence, 19a, and include or exclude an alternative spliced exon, 20a, respectively (Figure 5C). Both events result in p0071 protein isoforms with distinct C-terminal sequences including 1112 amino acids for p0071-3 and 1128 amino acids for p0071-4. The four p0071 protein isoforms include all ten armadillo ARM repeats. We also note the existence of complex alternative splicing and poly-adenylation of *delta-catenin* transcripts [57]. *p0071* mRNA expression analysis using primer combinations detecting the four putative *p0071* mRNA isoforms in MCF7 and MDA-MB-231 cells, together with a primer set spanning from exon 6 to exon 7 and accordingly envisaged to detect all the 3′-region variants of *p0071* (*p0071-pan*) mRNA, showed expression of all four isoforms and cell type dependent *p0071* alternative splicing and poly-adenylation (Figure 5D and data not shown). Exon 18 splicing to exon 19 compared with exon 18 splicing to exon 20 was relatively increased in MCF7 cells resulting in increased expression levels of the exon 19 including *p0071-1*, *p0071-3* and *p0071-4* mRNA isoforms (Figure 5D). We note that albeit our RT-qPCR analyses are not absolutely quantitative the analyses indicate that *p0071-1* and *p0071-2* mRNA are expressed in similar order of magnitudes. *p0071-3* mRNA is expressed to an approximately ten-fold lower level than *p0071-1* and *p0071-2* mRNA, and *p0071-4* mRNA is expressed approximately 40-fold lower, which is close to the limit of detection particular in MDA-MB-231 cells. Boyden chamber analysis showed that all *p0071* mRNA isoforms lacked significant protrusion localization in MCF7 cells (Figure 5B). In MDA-MB-231 cells *p0071-1* and *p0071-2* mRNA have protrusion localization ratios comparable with *p0071-pan* (Figure 5B). The *p0071-3* mRNA protrusion localization ratio was close to 1 indicating localization in neither MDA-MB-231 nor MCF7 cells (Figure 5B). *p0071-4* mRNA levels in MDA-MB-231 protrusions was limited to a level making absolute protrusion localization ratio calculations insignificant but indicates lack of localization (data not shown). Altogether, the *p0071* mRNA analyses indicate that protrusions localization requires 3′-UTR sequences present in exon 21 and that *p0071* mRNA protrusion localization have isoform and cell type specificity. The presented data are indicative of a model where localization of specific p0071 protein isoforms in MDA-MB-231 cells can be regulated at least partially through sub-cellular mRNA localization. If the observed *p0071* mRNA localization to protrusions in MDA-MB-231 cells is mechanistically linked to down-regulation of desmosomes and adherent junctions during EMT and thereby contributing to breast cancer invasion and metastasis requires further analysis [54].

## Conclusions

A limited amount of studies have addressed global-wide mRNA localization patterns in cancer cell protrusions. Here we show that a small size Boyden chamber assay can generate amounts of RNA from protrusions applicable for DRS. By this approach we identified hundreds of mRNA transcripts with localization in protrusions of metastatic breast cancer MDA-MB-231 cells. The presented approach can constitute an important experimental basis for additional studies to determine RNA localization as response to ECM-proteins and chemo-attractants. Moreover, the small amount of cellular material required for the DRS analyses will make the presented methodology highly applicable for studying the functional pathways mediating RNA localization using siRNA mediated depletion of candidate *trans*-factors and generation of cell lines with mRNA *cis*-element mutations. A more profound knowledge of the RNA localization to protrusions and how this localization is mediated can contribute considerable to the basic and clinical understanding of cancer cell metastasis.

## Competing interests

The authors declare that they have no competing interests.

## Authors’ contributions

KRJ, ES, RT and ALN designed the experiments. KRJ, ES, KKB and TFD carried out most of the experiments. KRJ, ES, KKB, TFD, RT and ALN participated in the interpretation of the data. ALN drafted the manuscript. All authors read and approved the final manuscript.

## Supplementary Material

Additional file 1: Table S1 List of primers used for PCR and RT-qPCR analysis. Click here for file

Additional file 2: Table S2 MDA-MB-231 boyden chamber DRS. Click here for file

Additional file 3: Table S3 Localized RNA (localization ratio above 1.6) in MDA-MB-231 protrusions compared with cell bodies. Click here for file
