# Supplementary material for: Direct RNA sequencing mediated identification of mRNA localized in protrusions of human MDA-MB-231 metastatic breast cancer cells
Source: J Mol Signal. 2013 Sep 1;8:9. doi: 10.1186/1750-2187-8-9 (PMC3844448; doi:10.1186/1750-2187-8-9)
Supplement: Additional file 1: Table S1 — List of primers used for PCR and RT-qPCR analysis. [file 1750-2187-8-9-S1.docx]

| **mRNA** | **Forward primer** | **Reverse Primer** |
| --- | --- | --- |
| ARPC3 | ACATGGCACTGTTGCCTATC | GAAGAAGACATTGGCCTTGA |
| ACTB | TCTACAATGGCTGCGTGTG | AGCCTGGATAGCAACGTACA |
| RAB13 | CCGCACTGTGGATATAGAGG | ATGGCTCCACGGTAGTAGG |
| KANK2 | TCTCACTCACAGATCGCGAT | GGATGATGTAGGGCTCTCGT |
| CORT | GTGAGAAGGAGCAAAGATGG | CATTCTCCCTCAGCTTGTGT |
| RHOA | GGAAGATTATGATCGCCTGA | GACTTCTGGGGTCCACTTTT |
| VIM | GACCAGCTAACCAACGACAAA | TGAAAGATTGCAGGGTGTTT |
| NET1 | GCTGGAGTACCTGGATGAAA | CCGAGTCAGAACCAAGATGT |
| ANP32B | CTGCCCCAGCTTACCTACTT | CTTCTTCACCATCCTCATCG |
| ZEB1 | AGACATGTGACGCAGTCTGGGT | TGGGCATTCATATGGCTTCTCTCCA |
| CENPB | CTCCACTTGGAACACGATCT | CAGCTTTGATGTCCAAGACC |
| SH3PXD2A | AAAGGATCATCCCCTTCCT | TCTTTTGGAGGGTTGACATC |
| TRAK2 | ATCTTTGGCAGCTGAGATTG | TGATGACACTTGAACGGTTG |
| PPFIA1 | ACAGATGGAGTGCTGGACAT | CACATGACTGGAAAGGGAAG |
| P0071-PAN | E6f, ACCTTCACTGCAAAGGACTG | E7r, CCCAAGCTGACTATGCTGAC |
| P0071-1 | E18f, ACCTGTGTCGACATTGGAGC | E19r, GGCATCCCCTTGGCTATTGT |
| P0071-2 | E18f, ACCTGTGTCGACATTGGAGC | E20E18r, GAAGGTTTGCTGGAGCCTGACTG |
| P0071-3 | E18f, ACCTGTGTCGACATTGGAGC | E19ar, CTGGCCAAGCGCCTGGAAATG |
| P0071-4 | E18f, ACCTGTGTCGACATTGGAGC | E20ar, GAGCTTGGTGTTGAAATGTAG |

List of primers used for PCR and RT-qPCR analysis,
